# Corneal Vibrations during Intraocular Pressure Measurement with an Air-Puff Method

**DOI:** 10.1155/2018/5705749

**Published:** 2018-02-11

**Authors:** Robert Koprowski, Sławomir Wilczyński

**Affiliations:** ^1^Department of Biomedical Computer Systems, University of Silesia in Katowice, Będzińska 39, 41-200 Sosnowiec, Poland; ^2^Department of Basic Biomedical Science, School of Pharmacy with the Division of Laboratory Medicine in Sosnowiec, Medical University of Silesia, Kasztanowa Street 3, 41-200 Sosnowiec, Poland

## Abstract

**Introduction:**

The paper presents a commentary on the method of analysis of corneal vibrations occurring during eye pressure measurements with air-puff tonometers, for example, Corvis. The presented definition and measurement method allow for the analysis of image sequences of eye responses—cornea deformation. In particular, the outer corneal contour and sclera fragments are analysed, and 3D reconstruction is performed.

**Methods:**

On this basis, well-known parameters such as eyeball reaction or corneal response are determined. The next steps of analysis allow for automatic and reproducible separation of four different corneal vibrations. These vibrations are associated with (1) the location of the maximum of cornea deformation; (2) the cutoff area measured in relation to the cornea in a steady state; (3) the maximum of peaks occurring between applanations; and (4) the other characteristic points of the corneal contour.

**Results:**

The results obtained enable (1) automatic determination of the amplitude of vibrations; (2) determination of the frequency of vibrations; and (3) determination of the correlation between the selected types of vibrations.

**Conclusions:**

These are diagnostic features that can be directly applied clinically for new and archived data.

## 1. Introduction

Currently, there exist numerous methods for measuring intraocular pressure. Some well-known methods use the Ocular Response Analyzer (ORA) [1] or Goldmann [2], Schiötz [3], or Corvis tonometers [4–9]. The last one, namely, the Corvis tonometer, uses an air puff in noncontact intraocular pressure measurements. Cornea deformation resulting from an air puff (and also the eyeball reaction), with simultaneous pressure measurement and registration using the ultra-high-speed Scheimpflug camera, enables determination of intraocular pressure (IOP). In addition, the use of the ultra-high-speed Scheimpflug camera allows for registration of deformation and thus the measurement of other additional features of the cornea. These features include the measurement of pachymetry, points and amplitude of applanation, or the maximum cornea deformation. These parameters are available in the original software (ver. 1.0) of the Corvis tonometer. In addition, with the newer software (ver. 1.1), it is possible to measure the eyeball reaction. These are numerical data or data in the form of two-dimensional graphs. The values are read using a slider, which also enables to observe a sequence of images illustrating successive stages of cornea deformation.

The ability to follow cornea deformation and obtain new additional parameters (except for IOP), such as the aforementioned applanation points, allows for a wide comparison of results obtained from different disease entities and with other tonometers.

So far, ophthalmologists have conducted a series of comparisons involving patients with keratoconus [10, 11] and diabetes [12–23], patients after surgery [24], and others [25–35]. In each case, only the parameters available in the original software of the Corvis tonometer were compared [5, 36–39]. The search for the terms “Corvis tonometer” or “cornea deformation” in the database of Authormapper articles provides the results presented in Table 1.

The data presented in Table 1 (possible multiple repetitions of the same publication) show that the area of cornea deformation analysis and attempts to connect the model with empirical results are still under investigation. The search for the same two keywords in the Web of Science database in terms of the number of citations provides the following results (see Table 2).

The summary presented in Table 2 clearly shows that the research related to the Corvis tonometer mostly involves clinical studies. These works are relatively new, covering a range of the past two years, and coincide with the development of the Corvis tonometer. On the other hand, publications on cornea deformation, including older studies, are most often cited. In the new studies relating to the Corvis tonometer, corneal vibrations were not analysed or only simulations were performed [62], despite the fact that their presence in image sequences was signalled in various papers [63–65]. The main obstacle was the lack of appropriate software or even image analysis algorithms allowing for their calculation or at least quantitative estimation. The first reports of corneal vibrations were presented in paper [66]. The first repeatable quantitative vibration analysis was reported in [67] and later extended in [68]. However, this is only one possible type of corneal vibration. For this reason, a wider analysis and definitions, including four different types of corneal vibrations occurring during IOP measurements by an air-puff method, are presented below.

## 2. Material

Input images were a sequence of 140 2D images of cornea deformation. The image resolution *M* × *N* = 200 × 576 pixels (where *M*—rows and *N*—columns) covered the area of 3.3 × 9.05 mm, which gave the pixel size of 16.5 × 15.7 *μ*m. A sequence of 140 images was acquired at discrete time points every 0.23 ms. No research or experiments on patients were performed as part of the study. The images were acquired (retrospectively) from the Corvis ST (OCULUS Optikgeräte GmbH, Germany) with software version 1.02, Image Database Repository, and artificially generated data (proprietary software in Matlab Version 7.11.0.584, R2010b) including healthy subjects. Proper operation of the algorithm was tested for tens of thousands of 2D images in Matlab Version 7.11.0.584, R2010b, with Image Acquisition Toolbox Version 4.0 and Image Processing Toolbox Version 7.1. There was no exclusion criterion—the proposed algorithm had to work correctly in all cases.

## 3. Method

**Figure 1:**
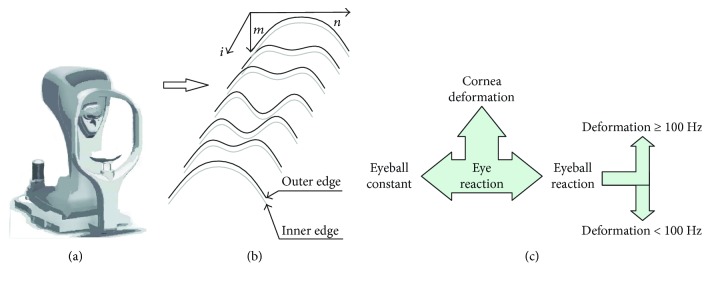
Block diagram of the subsequent major phases of measurement and analysis: (a) image acquisition using the Corvis tonometer, (b) automatic recording and analysis of the outer corneal contour, and (c) analysis allowing for the division of the eye reaction into three components and further separation of deformation with their respective frequencies.

The Corvis tonometer allows for the acquisition of data in different formats such as a video (^∗^.avi), a sequence of images (^∗^.jpg), or a losslessly compressed archive (^∗^.U12). One of the most convenient formats for analysis is a sequence of images ^∗^.jpg. Each measurement is stored as an image *L*_GRAY_(*m*,*n*,*i*) where *m*—row *m* ∈ (1,*M*), *n*—column *n* ∈ (1,*N*), and *i*—discrete time moments of measurement for which *i* = 1 for *t* = 0, *i* = 2 for *t* = 0.23 ms, *i* = 3 for *t* = 0.46, and so on. Each of the images *L*_GRAY_(*m*,*n*,*i*) is subjected to the outer corneal contour analysis (see Figure 1).

**Figure 2:**
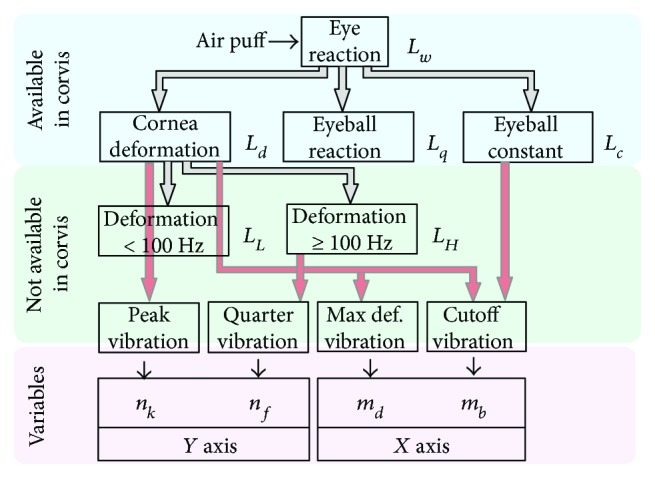
Block diagram of the division of image analysis into various stages and the adopted symbols.

As shown in paper [67], the best results were obtained for the dedicated contour detection algorithm. This algorithm provided better results than in the cases of the well-known Canny, Roberts, Prewitt, and Sobel filters [69–75]. Better results are herein understood as the results which provide the correctly detected corneal contour in a 10% greater number of cases. Operation of the contour detector presented in publication [67] is based on the sequential analysis of the cornea position for the next columns of the image *L*_GRAY_(*m*,*n*,*i*). This analysis enables automatic determination of the greatest object (cornea) and removal of noise, usually in the form of separate bright pixels. In addition, this operation enables the removal of uneven lighting. This is an extremely important feature of the algorithm because uneven brightness is significant for typical 2D cornea deformation images (*L*_GRAY_(*m*,*n*,*i*)). For this reason, typical and simpler tools of image analysis and processing such as binarization with a lower threshold, or binarization with two thresholds and an automatically selected threshold, for example, from the Otsu formula [76–79], cannot be used here. The outer corneal contour designated hereinafter as *L_w_*(*n*,*i*) was taken into consideration in the analysis. The results obtained, *L_w_*(*n*,*i*), were subjected to further processing: (1) cornea deformation—*L_d_*(*n*,*i*); (2) separation of the eyeball reaction—*Lq*(*n*,*i*); (3) designated corneal contour—*L_c_*(*n*,*i*); (4) deformations with a frequency of less than 100 Hz—*L_L_*(*n*,*i*); and (5) deformations with a frequency greater than 100 Hz—*L_H_*(*n*,*i*). The latter ones, that is, deformations with the frequency above 100 Hz, are the subject of further analysis. A block diagram of this division is shown in Figure 2.

Details of this known analysis stage are shown, for example, in paper [43], and will be omitted here. As is apparent from Figure 2, four different types of vibrations were separated from the waveforms *L_H_*(*n*,*i*), *L_c_*(*n*,*i*), and *L_d_*(*n*,*i*). They are described in detail in the following subsections.

### 3.1. First Type—Peak Vibration

Peak vibrations, variables *m_kl_* and *m_kr_*, are defined as the relative position changes in the row axis (*Y*) of the corneal contour local minima occurring between two applanations, *A*_*p*1_ and *A*_*p*2_. Their position for the subsequent time moments (parameter “*i*”) was calculated according to the following formula:

(1) $$\begin{array}{c} m_{kl}\left(i\right)=\underset{n\in \left(1,N/2\right)}{\min}\left(L_{d}\left(n,i\right)\right), \end{array}$$

where *i* ∈ (*A*_*p*1_, *A*_*p*2_).

Similarly,

(2) $$\begin{array}{c} m_{kr}\left(i\right)=\underset{n\in \left(N/2-1,N\right)}{\min}\left(L_{d}\left(n,i\right)\right), \end{array}$$

where *i* ∈ (*A*_*p*1_, *A*_*p*2_).

**Figure 3:**
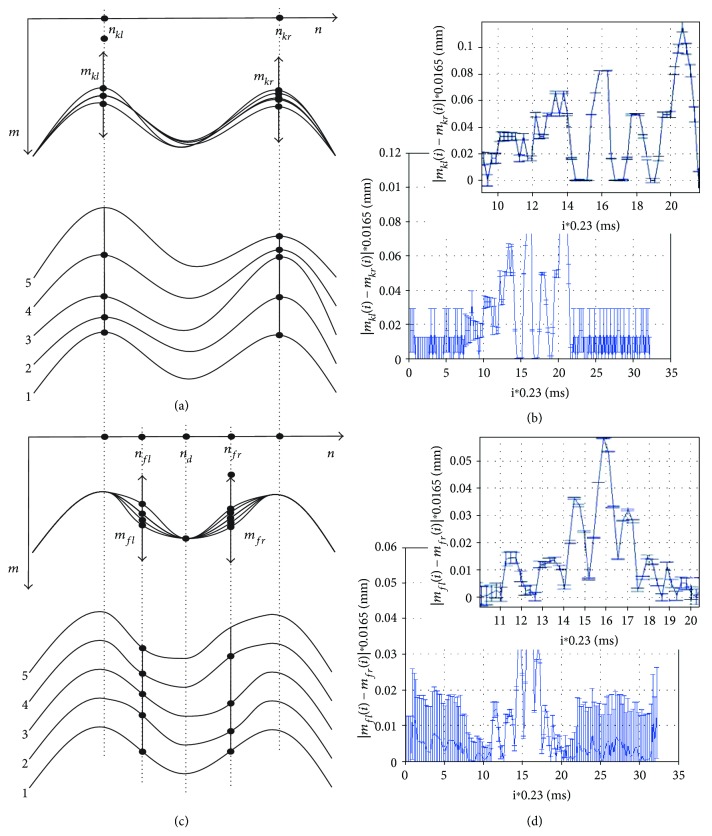
Diagram and results of peak vibration and quarter vibration calculations. (a, c) Schematic diagrams of calculating the peak vibration and quarter vibration and (b, d) examples of the results together with their magnification and measurement error *δ*, respectively. For the analysed case, *A*_*p*1_ = 10 ms and *A*_*p*2_ = 21 ms.

The minimum value is due to the adopted coordinate system referenced to the image (matrix) for which rows are numbered from the top starting from the first one. The measurement methodology is shown demonstratively for a sequence of five cornea deformation contours in Figure 3(a). Sample results, vibrations |*m_kl_*(*i*) − *m_kr_*(*i*)|, are shown as a function of time (consecutive images *i*) in Figure 3(b).

Values of the error *δ* visible on the graphs in Figures 3(b) and 3(d) result directly from the measurement idea. The error in determining the correct position of points *m_kl_*(*i*) and *m_kr_*(*i*) as well as *m_fl_*(*i*) and *m_fr_*(*i*) is strictly dependent on the amplitude of *L_d_*(*n*,*i*) (where *n* ∈ (1,*N*)). The greater the amplitude of *L_d_*(*n*,*i*), the greater the slope of the curves (Figures 3(a) and 3(c)) and the more accurate the measurement—subpixel accuracy. For the lack of cornea deformation (the end and beginning of measurement), this error is the greatest due to the largest signal-noise ratio—a maximum of ±1 pixel. It was estimated on the basis of research conducted in papers [45, 67, 68] that the error *δ* is approximately proportional to the amplitude of the waveform *L_d_*(*n*,*i*). Therefore, the measurement error *δ*, expressed in pixels, is calculated according to the following formula:

(3) $$\begin{array}{c} \delta \left(i\right)=\left\{\begin{array}{l} \frac{1}{\sum_{N}^{n=1}L_{db}\left(n,i\right)}\;\text{if }\overset{n=1}{\underset{N}{\sum }}L_{db}\left(n,i\right)\neq 0,\\ 1\;\text{if }\overset{n=1}{\underset{N}{\sum }}L_{db}\left(n,i\right)=0, \end{array}\right. \end{array}$$

where

(4) $$\begin{array}{c} L_{db}\left(n,i\right)=\left\{\begin{array}{l} 1\;\text{if }L_{d}\left(n,i\right)>P_{r},\\ 0\;\text{if }others, \end{array}\right. \end{array}$$

where *p_r_* is the binarization threshold determined at the level of 2 pixels taking into account the noise.

Figure 3(b) shows peak vibrations of all 140 registered images for the full time range and the magnification in the area covering the first and second applanations (*i* ∈ (*A*_*p*1_, *A*_*p*2_)). Peak vibrations, and to be more specific their absolute differences, range from 0 to 0.11 mm. Their largest amplitude is achievable a few milliseconds before the second applanation (*A*_*p*2_). By assessing peak vibrations as the absolute difference between *m_kl_*(*i*) and *m_kr_*(*i*), it is possible to become independent of the eyeball vibration or changes in its position relative to the tonometer. The measurement error range which is affected by the spatial image resolution error of 16.5 × 15.7 *μ*m and the adopted measurement method is marked on the graph in Figure 3(b). The larger the difference between *m_kl_*(*i*) and *m_kr_*(*i*), the smaller the measurement error.

### 3.2. Second Type—Quarter Vibration

Quarter vibrations, variables *m_fl_* and *m_fr_*, are defined as the relative position changes in the row axis of the two points of the corneal contour being in the middle of the distance between the peaks and the maximum deformation (*n_fl_*, *n_fr_*), measured for frequencies greater than 100 Hz. The vibrations of this type are most apparent in the qualitative evaluation of various stages of cornea deformation in the pressure measurement. The measured vibrations reach in these places the greatest amplitude [68]. The measurement of quarter vibrations was implemented according to the following formula:

(5) $$\begin{array}{c} m_{fl}\left(i\right)=L_{H}\left(\frac{n_{kl}+n_{d}}{2},i\right),\\ m_{fr}\left(i\right)=L_{H}\left(\frac{n_{kr}+n_{d}}{2},i\right), \end{array}$$

where *n_d_* is the maximum cornea deformation determined on the waveform *L_L_*(*n*,*i*) (see Figures 3(c) and 3(d)). The exact definition of finding points *n_d_* for the next *i* is presented in the next subsection. The obtained sample results of quarter vibrations (exactly |*m_fl_*(*i*) − *m_fr_*(*i*)|) are shown together with the measurement error in Figure 3(d). The maximum amplitude is equal to 60 *μ*m and occurs for the time moment (*i*) equal to 15.9 ms. It is worth noting that the maximum amplitude of quarter vibrations occurs in between two applanations *A*_*p*1_ and *A*_*p*2_.

### 3.3. Third Type—Maximum Deformation Vibration

The maximum deformation vibration, variable *n_d_*, is defined as the absolute changes in the position of the local maximum in the column axis (*X*). The position of the local maximum is determined at applanation intervals (*i* ∈ (*A*_*p*1_, *A*_*p*2_)) and *n_d_* ∈ (*n_fl_*, *n_fr_*), that is,

(6) $$\begin{array}{c} n_{d}\left(i\right)=\underset{n\in \left(n_{fl},n_{fr}\right)}{\arg\max}\left(L_{d}\left(n,i\right)\right). \end{array}$$

**Figure 4:**
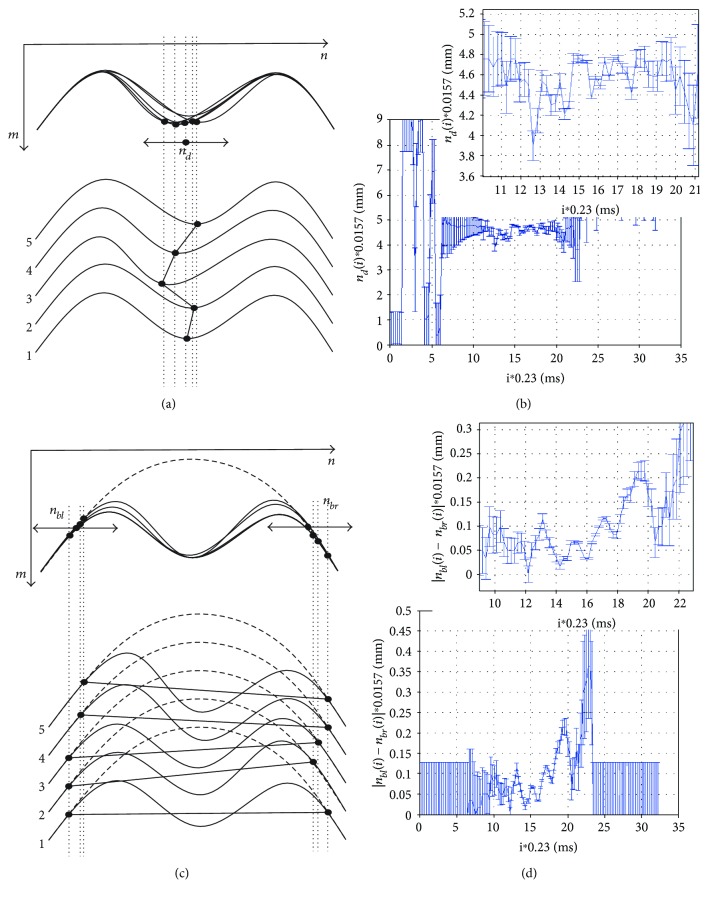
Diagram and results of calculating the maximum deformation vibration and cutoff vibration. (a, c) Schematic diagrams of calculating the maximum deformation vibration and cutoff vibration and (b, d) examples of the results obtained together with their magnification and measurement error, respectively.

Figures 4(a) and 4(b) show graphically measurement ideas and examples of vibration analyses. The graphs in Figures 4(b) and 4(d) show examples of results of *n_d_*(i), absolute differences between *n_bl_*(*i*) *− n_br_*(*i*), and the values of the measurement error calculated according to (4).

The maximum deformation vibration analysis provides quite interesting results concerning the movements of the maximum cornea deformation during measurement. These movements are in the range of 0.3 mm, and for all the analysed cases, they reach a greater value before the second applanation *A*_*p*2_ (20, 21 ms).

### 3.4. Fourth Type—Cutoff Vibration

Cutoff vibrations—*n_bl_* and *n_br_*—are defined as the relative position changes in the column axis (*X*) of the end points in which the cornea changed its position with respect to the original position (see Figure 4(c)).

(7) $$\begin{array}{c} n_{bl}\left(i\right)=\underset{n\in \left(1,N/2\right)}{\min}\left(\left|L_{d}\left(n,i\right)-L_{d}\left(n,1\right)\right|\right), \end{array}$$

(8) $$\begin{array}{c} n_{br}\left(i\right)=\underset{n\in \left(N/2+1,N\right)}{\min}\left(\left|L_{d}\left(n,i\right)-L_{d}\left(n,1\right)\right|\right), \end{array}$$

where *i* ≠ 1.

**Figure 5:**
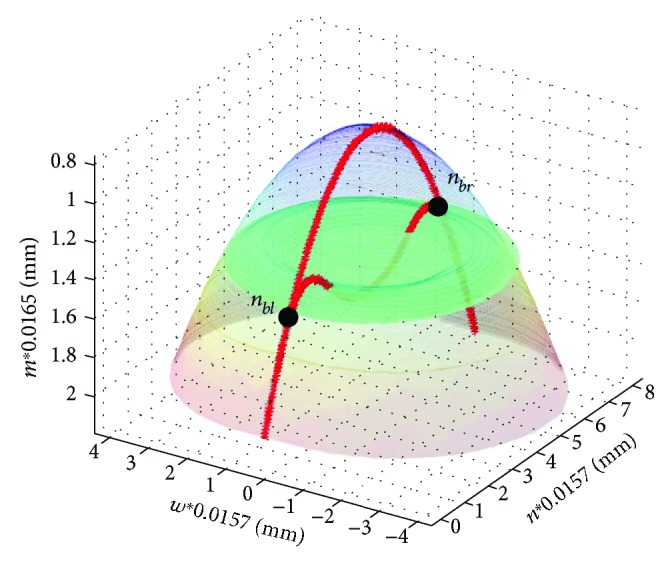
Three-dimensional reconstruction of cornea deformation, the outer contour, for *t* = 0 ms (blue) and 2.31 ms (brown) with marked points *n_bl_* and *n_br_* (green plane). The outer contour for *t* = 0 ms and 2.31 ms being the basis for calculation is marked in red.

Equations (7) and (8) relate to the last and first detected minimum. The obtained measurement results |*n_bl_*(*i*) *− n_br_*(*i*)| (Figure 4(d)) confirm the cutoff vibration change of 0.2, 0.3 mm. The locations of points *n_bl_* and *n_br_* are also shown on the 3D reconstruction, in Figure 5, which can be performed by using basic data concerning biomechanics of the cornea (mainly viscoelasticity) and its possible deformations [42, 44, 61].

The reconstruction shown in Figure 5 was performed using the linear interpolation for the next points of the corneal contour spaced with respect to its main axis of symmetry (an additional dimension “*w*” was created in this way).

**Figure 6:**
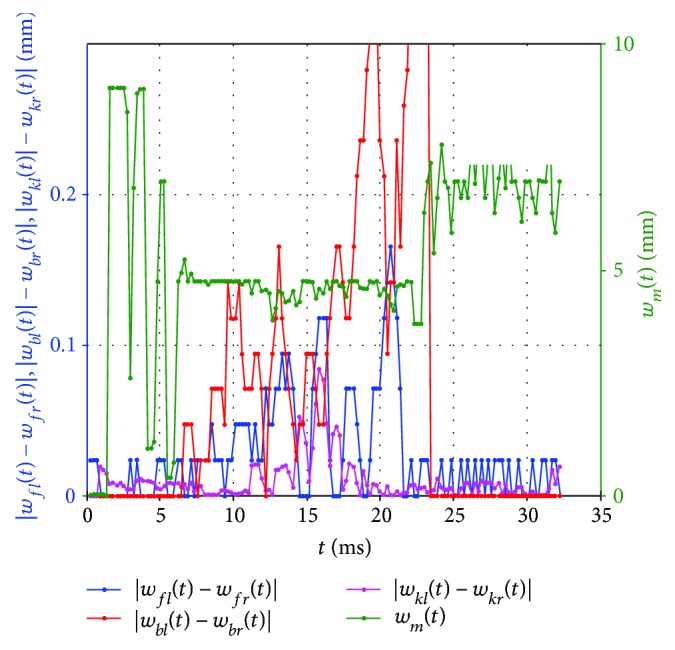
Summary graph of four different types of corneal vibrations.

The summary of four different types of vibrations is shown in Figure 6.

## 4. Discussion

The four types of corneal vibrations occurring during IOP measurement form the basis for further analysis. The assessment of clinical usefulness is related to different vibration types with varying degrees.

### 4.1. Cutoff Vibrations

Vibrations of the start and end points of cornea deformation are associated with the lack of symmetry in deformation. In addition, large values of the cutoff vibration result from the fact that measurements are performed near the first or second applanation. In near applanation (*A*_*p*1_, *A*_*p*2_), the cornea is flattened and calculations are less accurate. Vibrations of this type are the least resistant to noise and artefacts occurring in the sequence of analysed images.

### 4.2. Maximum Deformation Vibration

This vibration is essential for the determination of the maximum deflection waveform as a function of time. Waveforms of such a function are shown by default in the Corvis tonometer software. However, the graphs do not enable to correct the position change (vibration) of the maximum deformation point in the column axis (*X*). The graphs provided in the Corvis software relate to the analysis of movement in the row axis (*Y*) of only one point—usually the main axis of the cornea.

### 4.3. Quarter Vibration

**Figure 7:**
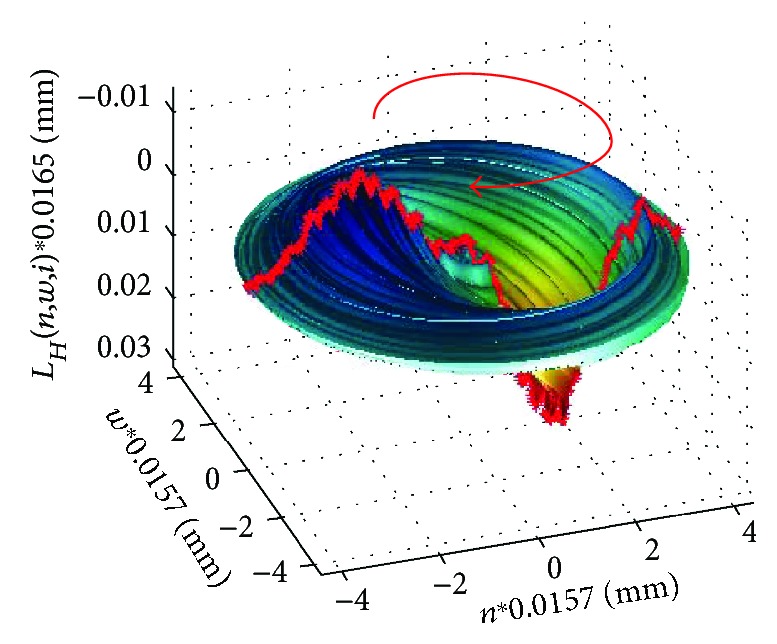
3D reconstruction of corneal vibrations for *t* = 14.5 ms. The measured area and conventionally adopted vibration direction are marked in red.

**Figure 8:**
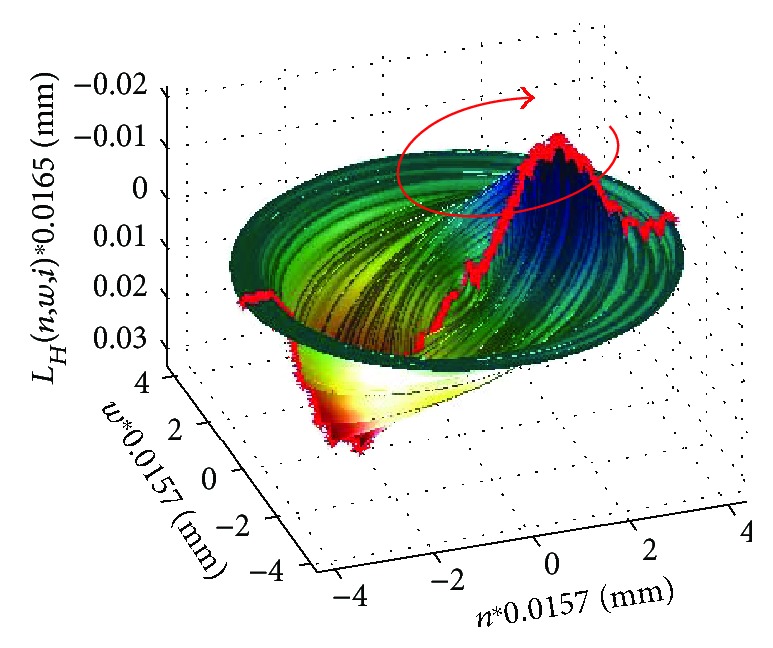
3D reconstruction of corneal vibrations for *t* = 15.9 ms. The measured area and conventionally adopted vibration direction are marked in red.

As mentioned above, it is the most common vibration type occurring in literature [43, 45, 65, 66, 68]. However, it is nowhere precisely defined. Vibrations of this type result from the wave phenomena occurring in the eye with the vitreous. Figures 7 and 8 show a 3D reconstruction of corneal vibrations for the selected time moments *t* = 14.5 ms and 15.9 ms as well as the minimum and maximum marked in red, which form the basis for calculating quarter vibrations.

The vibration directions indicated with red arrows in Figures 7 and 8 were adopted conventionally. When analysing quarter vibrations, it is possible to notice cyclical movements of the points *m_fl_* and *m_fr_* from the maximum to the minimum vibration amplitude, respectively—in this case, ±0.03 mm. The points (*m_fl_*,*m_fr_*) together with the points (*n_fl_*,*n_fr_*) (Figure 3(a)) form, at each time moment, the angle *λ*, that is,

(9) $$\begin{array}{c} \lambda \left(i\right)=a\tan2\left(m_{fl}\left(i\right)-m_{fr}\left(i\right),n_{fl}\left(i\right)-n_{fr}\left(i\right)\right), \end{array}$$

where *a*tan2 is the four-quadrant inverse tangent (arctangent) of the real parts of *m_fl_*(*i*) − *m_fr_*(*i*) and *n_fl_*(*i*) − *n_fr_*(*i*).

The sum of the angular values *λ*(*i*) at each *i*th time moment enables to determine the angle *β*(*i*):

(10) $$\begin{array}{c} \beta \left(i\right)=\overset{i}{\underset{i=1}{\sum }}\lambda \left(i\right). \end{array}$$

**Figure 9:**
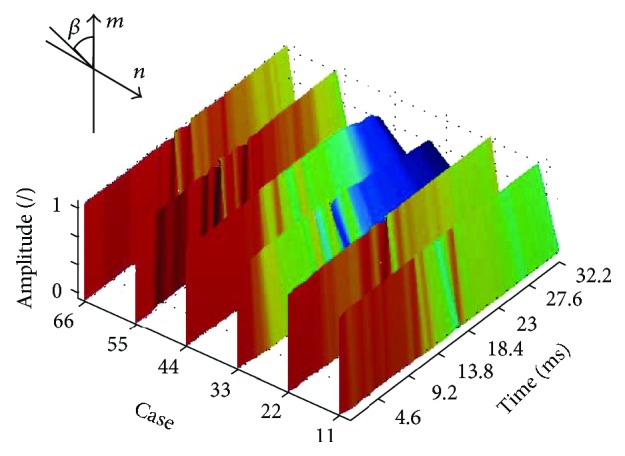
Changes in the angle *β*(*i*) for the quarter vibration for the first six patients (normalized amplitude). The graph is shown for the artificial colour palette.

**Figure 10:**
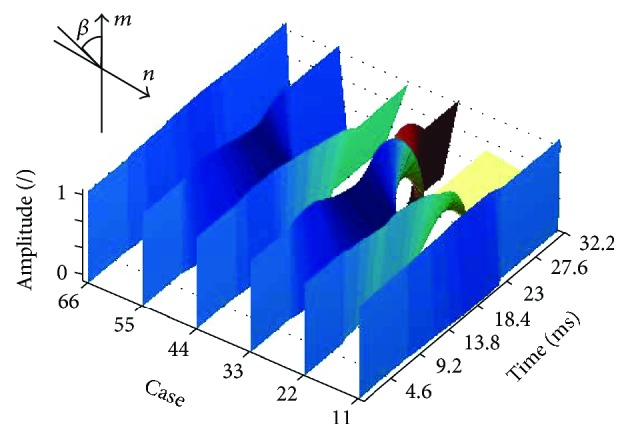
Changes in the angle *β*(*i*) for the peak vibration for the first six patients (normalized amplitude). The graph is shown for the artificial colour palette.

The changes in the value of the angle *β*(*i*) for the quarter vibration and peak vibration are shown in Figures 9 and 10.

As is apparent from the presented graphs (Figure 9), the values of the angle *β*(*i*) for the quarter vibration do not exceed 90° in any of the six cases. This is due to the relatively small amplitude of the quarter vibration (±0.03 mm).

### 4.4. Peak Vibrations

These are vibrations having much larger amplitude and values of the angle *β*(*i*) than quarter vibrations (Figure 10). Peak vibrations are a consequence of quarter vibrations—correlation for all the analysed cases was 0.78. Peak vibrations are very insensitive to the noise occurring in images. At any time moment, determination of the local minimum values in accordance with the definition is algorithmically clearly defined and fairly simple.

The frequency of all four vibration types and their simplified definitions are given in Table 3.

The results presented in Table 3 are sensitive to changing parameters in different ways. The most sensitive to changing parameters is the cutoff vibration. This is due to the idea of measurement, in which the most sensitive element is designation of points *n_bl_* and *n_br_*—the junction of the original (steady) state of the corneal contour with the analysed contour. For this reason, there are large discrepancies in determining the vibration frequency (302 ± 112 Hz). The results obtained for the other vibration types are less dependent on the selection of the parameters and are limited to the resolution error of around ±17 *μ*m. As noted earlier, the peak vibration is the least sensitive to changing parameters and noise. The individual harmonics of the selected vibration type are similar to each other in terms of both frequency and amplitude (areas of flat frequency spectrum). For this reason, the vibration amplitude (of any of the four discussed vibrations) calculated in accordance with the proposed definition is several times greater than the amplitude of the first harmonic. The recorded vibrations consist of several harmonics whose amplitude is not too large (single pixels). The vibration waveform analysis for all components with higher (>100 Hz) frequency not only enables to reduce the measurement error but also ensures better reproducibility of measurements for a single patient.

The presented types of corneal vibration can be analysed in terms of their diagnostic clinical usefulness. For example, for such defined vibrations, a comparative analysis with patients with keratoconus or glaucoma can be performed. Any change in the biomechanical conditions of the cornea and in particular corneal thickness [32, 80–86] affects the measured biomechanical parameters of vibrations. Evaluation of the effect of these diseases on the obtained results and their diagnostic usefulness will be the subject of subsequent authors' papers.

## 5. Critical Summary

This paper proposed and defined the measurement method and presented the results for four different types of corneal vibrations based on the literature review. The proposed measurement method has the following advantages:

- The measured vibrations are a new feature (actually a set of four features) complementing other known characteristics measured using the Corvis tonometer (such as applanation times, pachymetry, or IOP).
- Definitions of the various vibrations are easy to record, which facilitates their physical interpretation and assessment of their clinical application.
- Vibration measurement can be performed based on the image sequences from the ultra-high-speed Scheimpflug camera during pressure measurement with an air-puff method using any other device (not just the Corvis tonometer).
- All measurements of vibrations are fully reproducible and provide the results in a fully automated way.
- Vibration analysis time on the PC with the Intel Core i7-4960X CPU @ 3.60 GHz is a few seconds for a new sequence of analysed images and less than a second when reading the corneal contour data matrix from the hard disk.
- Cutoff vibration measurement is most critical in terms of the sensitivity to changing parameters.
- There is a statistically significant correlation between peak vibration and quarter vibration.

The results can be compared with a number of works based on the corneal vibration simulation. In paper [64], the authors demonstrated that optical coherence tomographic (OCT) vibrography is able to determine corneal material parameters, while reducing current prevalent restrictions of other techniques (such as intraocular pressure (IOP) and thickness dependency). Results from the simulation can be successfully compared with the obtained results of vibration measurements proposed in this article. Similarly, in the doctoral thesis [87], there is a significant discrepancy between the results obtained from simulation and the ones obtained in practice. The vibration analysis presented in publication [68] concerns only one of the described vibration types (quarter vibration), and only the first harmonic is analysed. Therefore, in the subsequent papers, the authors intend to conduct detailed clinical studies comparing the amplitude and frequency of the various types of vibration for different diseases: keratoconus, peripheral blood pressure, diabetes, operating fugitives, and so on. Repeatability of measurements of the defined four vibration types and, in particular, the impact of individual variability of patients will be also measured. In addition, there will be an attempt to carry out 3D modelling of vibrations using, for example, the finite element method.

The results presented in this article may also be compared with the results obtained by other authors. This type of comparison concerns two areas of articles: for which the results are obtained from actual measurements (healthy subjects and ill patients), for which the results are obtained from simulations. In the first case, the results of other authors, Mercer et al. [88], relate to cornea deformation analysis during intraocular pressure measurement of 89 eyes (47 normal, 42 keratoconic) using the Corvis tonometer and a validation arm of 72 eyes (33 normal, 39 keratoconic) using the Corvis ST. Keratoconus was diagnosed by clinical findings and confirmed by topography and tomography. In turn, Jung et al. [89] analysed 75 healthy subjects and 136 patients from a glaucoma group. After adjusting potential confounding factors, including the intraocular pressure, age, central corneal thickness, and axial length, the deformation amplitude was smaller in the glaucoma group (1.09 ± 0.02 mm) than in the normal control group (1.12 ± 0.02 mm) for *p* = 0.031. According to the results provided by other authors [89], the deformation amplitude and the deflection amplitude of the severe glaucoma group (1.12 ± 0.02 mm and 0.92 ± 0.01 mm) were significantly higher than in the case of the early glaucoma group (1.07 ± 0.01 mm and 0.88 ± 0.11 mm), *p* = 0.006 and *p* = 0.031, respectively, whereas for the moderate glaucoma group (1.09 ± 0.02 mm and 0.90 ± 0.02 mm), they were greater than for the early glaucoma group, but this difference was not statistically significant. Very interesting findings were presented by Boszczyk et al. [90], who analysed 10 patients in whom biomechanical parameters of the cornea were measured during IOP measurement. The authors showed that intraocular pressure and amplitude of corneal indentation are inversely related (*p* = 0.0029), but the correlation between intraocular pressure and amplitude of eye retraction is low and insignificant (*p* = 0.51).

The results of simulation and application of phantoms are another analysed area. In [91], Bekesi et al. analysed a new method for reconstructing corneal biomechanical properties from air puff cornea deformation images with the use of hydrogel polymer model corneas and porcine corneas. The simulated stress-strain curves of the studied hydrogel corneal materials fitted the experimental stress-strain curves from uniaxial extensiometry well, especially in the 0–0.4 range. The equivalent Young's moduli of the reconstructed material properties for the three polymer materials were 0.31, 0.58, and 0.48 MPa and differed by <1% from those obtained from extensiometry. Unfortunately, this article [91] did not attempt to analyse the vibration of the corneal model during deformation. Similarly, Elham et al. [92] did not analyse vibrations, but only the basic parameters of the cornea. The obtained results concern 10 compared parameters, and the means of 8 were significantly different between groups (*p* < 0.05 – 48 keratoconic eyes were compared with the corresponding ones in 50 normal eyes). The means of the parameters did not show significant differences between keratoconus subgroups. In [93], the authors propose a laboratory corneal model that was subjected to various pressures and thermal and mechanical factors in order to better understand the genesis of keratoconus deformations. An interesting study was published by Matalia et al. [94]. It related to the analysis of the correlation between corneal biomechanical stiffness and refractive error (RE) in the paediatric population. 733 thoroughly examined paediatric eyes were included in the study retrospectively. However, this work only refers to the results of simulations. Matalia et al. [94] have confirmed the usefulness of high-field MRI in understanding ocular biomechanics. They created a very interesting model of the eye to explain the occurring biomechanical processes. Due to the high-field MRI limitations, this study does not include corneal vibration analysis during intraocular pressure measurement.

The above summary shows that the analysis of corneal vibration during intraocular pressure measurement using an air puff represents the future for both modelling and phantoms [95–97] as well as for the analysis of patients and healthy subjects.

The well-known software in the Corvis tonometer (OCULUS Optikgeräte GmbH, Germany, software version 1.02) can analyse the basic, above-discussed biomechanical parameters of the cornea, but it does not analyse corneal vibrations [67, 68, 98, 99]. Thus, it leaves open space for researchers dealing with this field of knowledge.

In subsequent studies, the authors intend to use the discussed biomechanical parameters of the cornea and in particular its vibration for the diagnosis of such diseases as diabetes, keratoconus, or glaucoma. Preliminary analysis and comparison encourage further research and measurements in this area. So far, they have been carried out without introducing the definitions discussed in this article, thus making it difficult to compare and establish diagnostic significance, for example, in the diagnosis of keratoconus.

## Figures and Tables

**Table 1:** Summary of the first three results in the Authormapper database for the terms “Corvis tonometer” and “cornea deformation” as of October 16, 2017.

Term	Country/unit/author		Number of publications	Publications
Corvis tonometer	Country	Germany	6	[40–42]
		Poland	17	[43–45]
		Israel	1	[46]
	Unit	University of Silesia	13	[44, 45]
		Care-Vision Hamburg	1	[10]
		Medical University of Silesia	1	[43]
	Author	Koprowski R.	17	[43–45]
		Druchkiv V.	2	[10, 41]
		Frings A.	2	[10, 41]

Cornea deformation	Country	Poland	27	[43–45]
		United States	370	[46–48]
		South Korea	21	[49–51]
	Unit	University of Zielona Gora	506^∗^	[52]
		NanoEnTek Inc. & Digital Bio Technology Co. Inc.	225^∗^	[53]
	Author	Koprowski R.	12	[54]
		Elsheikh, Ahmed	8	[55]
		Grishin, V. N.	7	[56]

^∗^Possible multiple repetitions of the same publication.

**Table 2:** Summary of the first three publications with the highest number of citations in the Web of Science database for the terms “Corvis tonometer” and “cornea deformation” as of October 16, 2017.

Term	Year	Publication	Number of citations	Number of analysed patients
Corvis tonometer	2013	Hong J. X. [57]	69	36 ill patients, 23 healthy subjects
	2013	Reznicek L. [58]	40	142 eyes with glaucoma and 36 control eyes
	2014	Ali N. Q. [59]	38	103 healthy eyes

Cornea deformation	2005	Storm C. [60]	739	None
	2005	Liu J. [61]	370	Model
	2006	Congdon N. G. [62]	257	230 subjects, 194 had a diagnosis of primary open-angle glaucoma

**Table 3:** Summary of the results of calculating mean frequency of the fundamental harmonic and the amplitude of the discussed vibration types (and their standard deviation of the mean) for 10 cases (for healthy subjects) occurring between applanations.

Vibration type	Measurement in the axis	Definition	Frequency	Vibration amplitude (all harmonics)
Cutoff vibration	*X*	|*n_bl_*(*i*) *− n_br_*(*i*)|	302 ± 112 Hz	0.37 ± 0.11 mm
Maximum deformation vibration	*X*	*n_d_*(*i*)	515 ± 99 Hz	0.54 ± 0.21 mm
Quarter vibration	*Y*	|*m_fl_*(*i*) − *m_fr_*(*i*)|	408 ± 68 Hz	74.5 ± 14 *μ*m
Peak vibration	*Y*	|*m_kl_*(*i*) − *m_kr_*(*i*)|	401 ± 59 Hz	93.2 ± 20.4 *μ*m
